# Biofluorescent response in lumpfish *Cyclopterus lumpus* to a therapeutic stressor as assessed by hyperspectral imaging

**DOI:** 10.1038/s41598-024-53562-7

**Published:** 2024-02-05

**Authors:** Thomas Juhasz-Dora, Stein-Kato Lindberg, Amanda Karlsen, Samuel Ortega

**Affiliations:** 1 Bantry Marine Research Station, Bantry, P75 AX07 Ireland; 2 School of Biological, Earth and Environmental Sciences, University College Cork, Cork, T23 N73K Ireland; 3 Nofima AS, 9291 Tromsø, Norway

**Keywords:** Biological fluorescence, Ichthyology

## Abstract

The demand for lumpfish (*Cyclopterus lumpus*) as a biological control for salmon lice is increasing. However, lumpfish welfare is considered a limiting factor within aquaculture operations. Identifying a noninvasive parameter that measures subclinical stress in lumpfish is a key goal for improving their welfare. The lumpfish is documented to emit green and red biofluorescence within the blue shifted light of their environment. Here we show that lumpfish fluorescence responds to a therapeutic stressor within a controlled experiment. Lumpfish (n = 60) underwent a 3-h freshwater bath therapeutant to evaluate whether fluorescence spectra produced by the species respond to external stimuli. Lumpfish were quickly scanned under a hyperspectral camera (400–1000 nm spectral range) prior to and after treatment. The lumpfish were randomly divided into 3 groups with identical treatment. All groups increased fluorescence emissions, though the level of change depended on whether the averaged, red, or green spectra were analyzed; the control group (n = 20) remained constant. All lumpfish emitted green fluorescence (~ 590–670 nm) while a portion (49%) produced red fluorescence (~ 690–800 nm). As lumpfish fluorescence shifts in response to the applied stressor, this study provides insight into how fluorescence may be incorporated into the welfare management of lumpfish.

## Introduction

The market for lumpfish (*Cyclopterus lumpus* 1758 Linnaeus) to control sea lice in salmon farms is rising due to increasing resistance of *Lepeophtheirus* lice to licensed therapeutants^1^. Sea lice infestations in Atlantic salmon farms significantly impact the welfare and productivity of farmed fish, causing substantial economic losses where densities reach high levels^2^. However, lumpfish welfare concerns, such as systemic infections, are considered a limiting factor within aquaculture operations for incorporating this species to combat sea lice^3^. The difficulty of identifying parameters which successfully link together husbandry, health, and allostatic responses to environmental factors has slowed the formation of operational welfare indicators (OWIs) that can be used as an overall application for this species. A protocol developed by Eliasen et al.^1^ to address this issue focused on the weight/length relationship, external physical damage scores, stomach content analysis and calibrated liver color scores. Though useful, this OWI remains a representative sampling technique of a population that requires invasive (lethal) techniques. As lumpfish are typically kept in high densities during the hatchery and sea pen deployment stages, developing a non-contact method to monitor lumpfish welfare in production is needed.

The therapeutic stressor chosen for this study is based on an accepted veterinary therapeutant (freshwater bath) to control the amoebic gill disease (AGD) pathogen *Paramoeba perurans* in lumpfish aquaculture operations^4^. The standard therapy for AGD in lumpfish is a 3-h freshwater bath, with a salinity of less than three parts per thousand (ppt)^5^. The histological results, good tolerance of lumpfish to freshwater, and the lack of morbidity/post treatment mortalities during clinical trials indicate that treatment of lumpfish with freshwater baths can be successful with either short- or long-term treatments^4^. Juvenile lumpfish reared in an aquaculture facility are known to produce a bright green fluorescence^6^. Furthermore, a study of lumpfish serum taken from sexually mature wild fish found female lumpfish producing a blue–green blood serum that fluoresced a pale blue under long-wave UV light (~ 350 nm), with the red-colored male serum emitting a magenta–orange fluorescence^7^. This difference in fluorescence is due to the sexual dimorphism within the species, with smaller (30 ± 10 cm) males exhibiting orange–red spawning coloration while the rotund (42 ± 10 cm) females show a blue–green color^8^. Biofluorescence is a well-documented phenomenon in a broad assemblage of fish orders^9,10^. For example, the closely related Pacific spiny lumpsucker *Eumicrotremus orbis* emits fluorescence that is sexually dimorphic, with males producing red whilst females emit green fluorescence^11^. Other members of the Scorpaeniformes such as the tropical Barchin stonefish *Sebastapistes strongia*^9^ and the cold water variegated snailfish *Liparis gibbus*^12^ were documented to produce fluorescent emissions in both red and green spectra, suggesting that dimorphism in fluorescence may be widespread. As lumpfish are regularly exposed to stress through hatchery and sea deployment operations^13^, we hypothesize that lumpfish biofluorescence responds to stressors in their environment.

Hyperspectral imaging technology has the capacity to provide a non-destructive method to monitor changing parameters within live fish that includes fluorescence detection. Detailed spectral bands of emitted light between 400 and 1000 nm spectral range captured by hyperspectral imaging exceed the recording capacity of traditional cameras^14^. The light measured for each pixel is evenly sampled with high resolution across the electromagnetic spectrum, and the resulting signal depends on the chemical and physical composition of the imaged object^15–17^. Available publications on the use of hyperspectral imaging on live fish are limited. However, scientists have utilized reflectance spectroscopic measurements for the characterization of camouflaging patterns of various marine species^18,19^. Additionally, hyperspectral imaging has been employed to identify the smoltification status in Atlantic salmon^20,21^. Further studies used freshly euthanized Atlantic salmon smolts in successfully auditing hemorrhaging in the dorsal fin region^22^.

The objectives of our study were: (1) to evaluate whether hyperspectral imaging can effectively monitor biofluorescence in live lumpfish, and (2) to investigate whether lumpfish fluorescence responds as a group to the therapeutic stressor.

## Results

### General fluorescence

**Figure 1:**
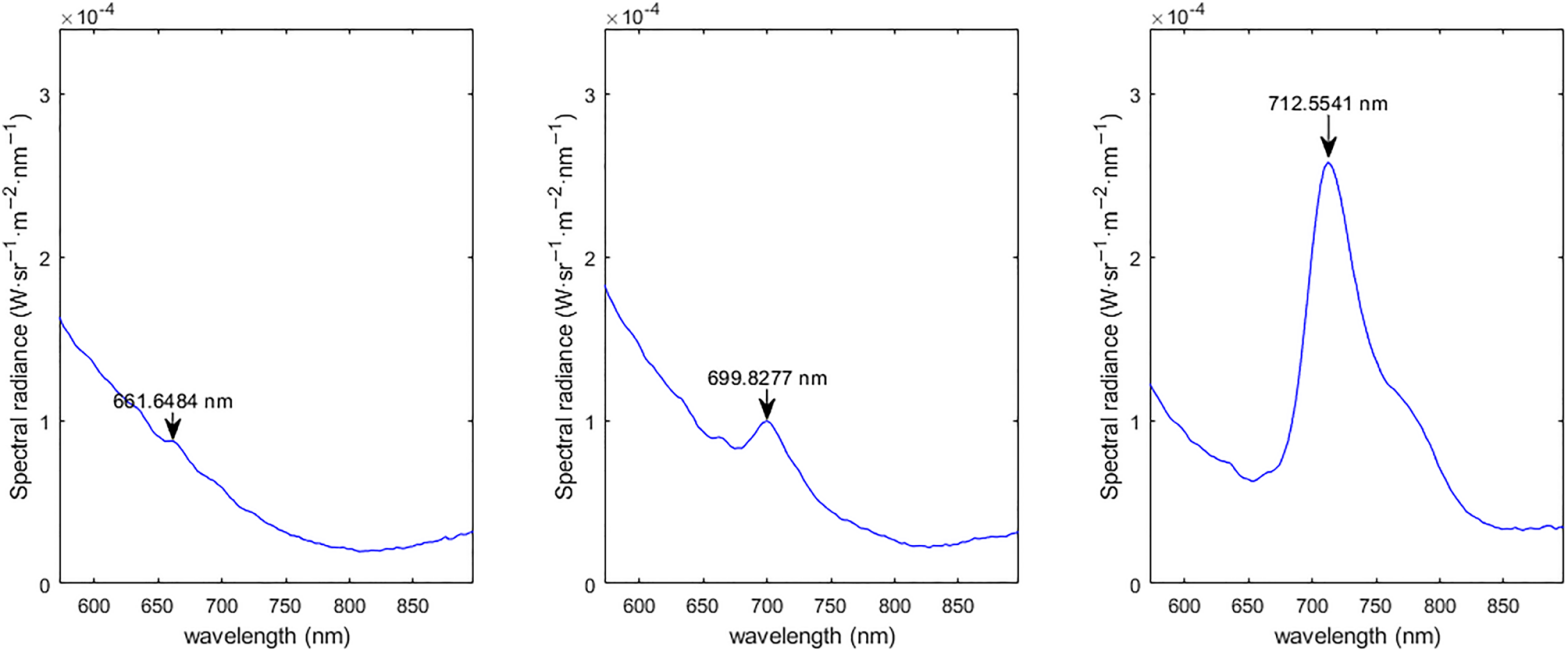
Variations in intraspecific fluorescence in lumpfish: dominant green spectra (**a**) and dominant red spectra (**b**,**c**). The figure displays the average fluorescence spectra from representative fish examples in the dataset exhibiting distinct fluorescence characteristics.

**Figure 2:**
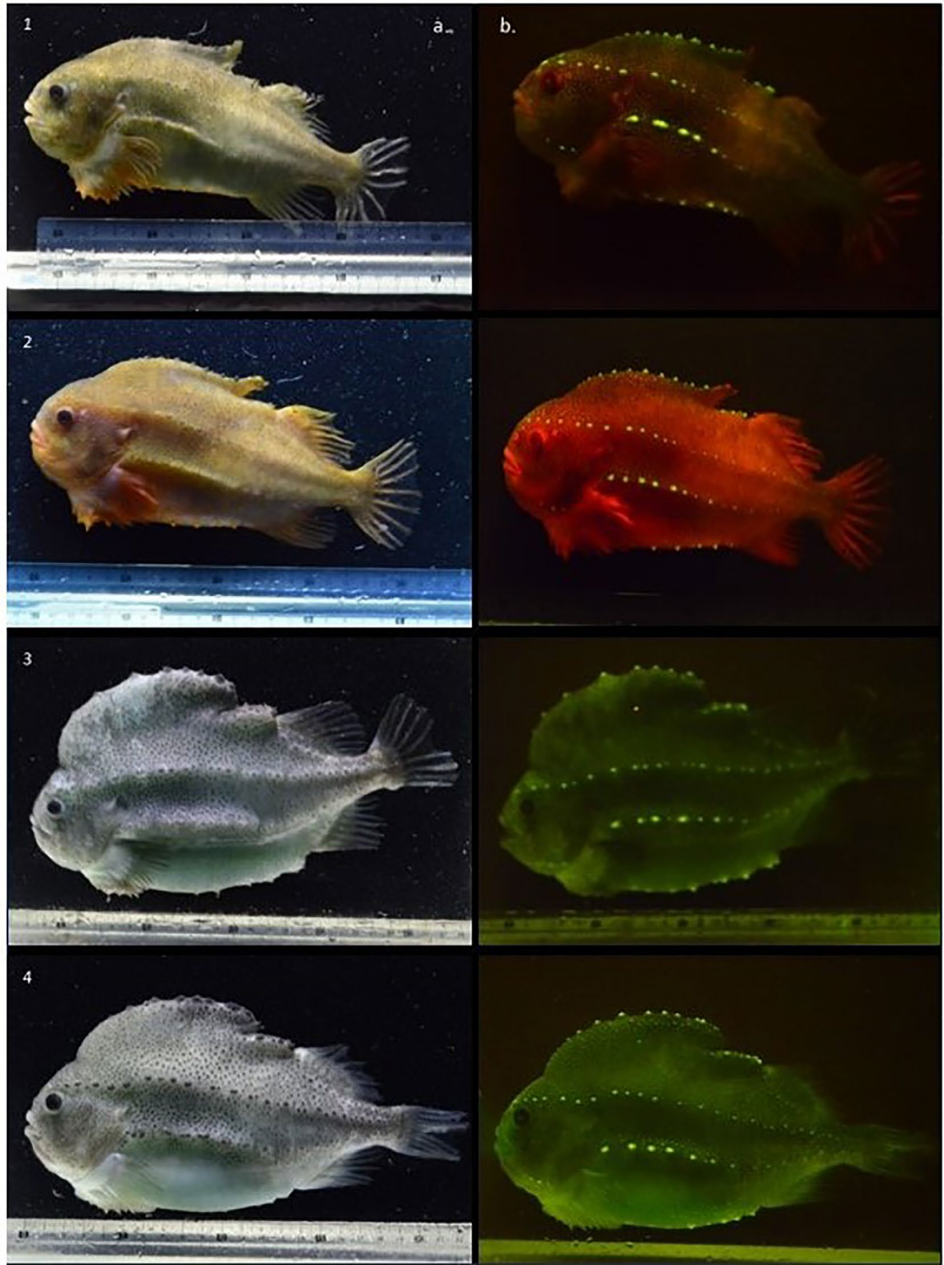
Red, green, and blue** (**RGB) photography taken for illustrative purposes of male (1,2) and female lumpfish (3,4) under ambient white lighting (**a**) and royal blue excitation lighting (~ 445 nm) (**b**). Male lumpfish express a red fluorescence through the body, with green fluorescence emissions from the tubercles lining the modified dorsal fin and longitudinal body lines. The green fluorescence produced by female lumpfish are concentrated within the tubercules.

The lumpfish within this study produced 2 distinct fluorescence peaks, a broad green peak (~ 580–690 nm), and a pronounced red emission maximum (~ 690–715 nm). The documented fluorescence spectra are defined as green dominant with only the broad green peak fluorescence and red dominant that emits both the green fluorescence and a sharp emission peak in the red spectra as seen in Fig. 1. Half of the measured lumpfish (n = 80) have the red dominant spectra (49%), while the remaining have the green dominant spectra (51%). A visual representation of the red and green fluorescence produced by lumpfish can be seen in Fig. 2.

### Fluorescence spectra analysis

**Figure 3:**
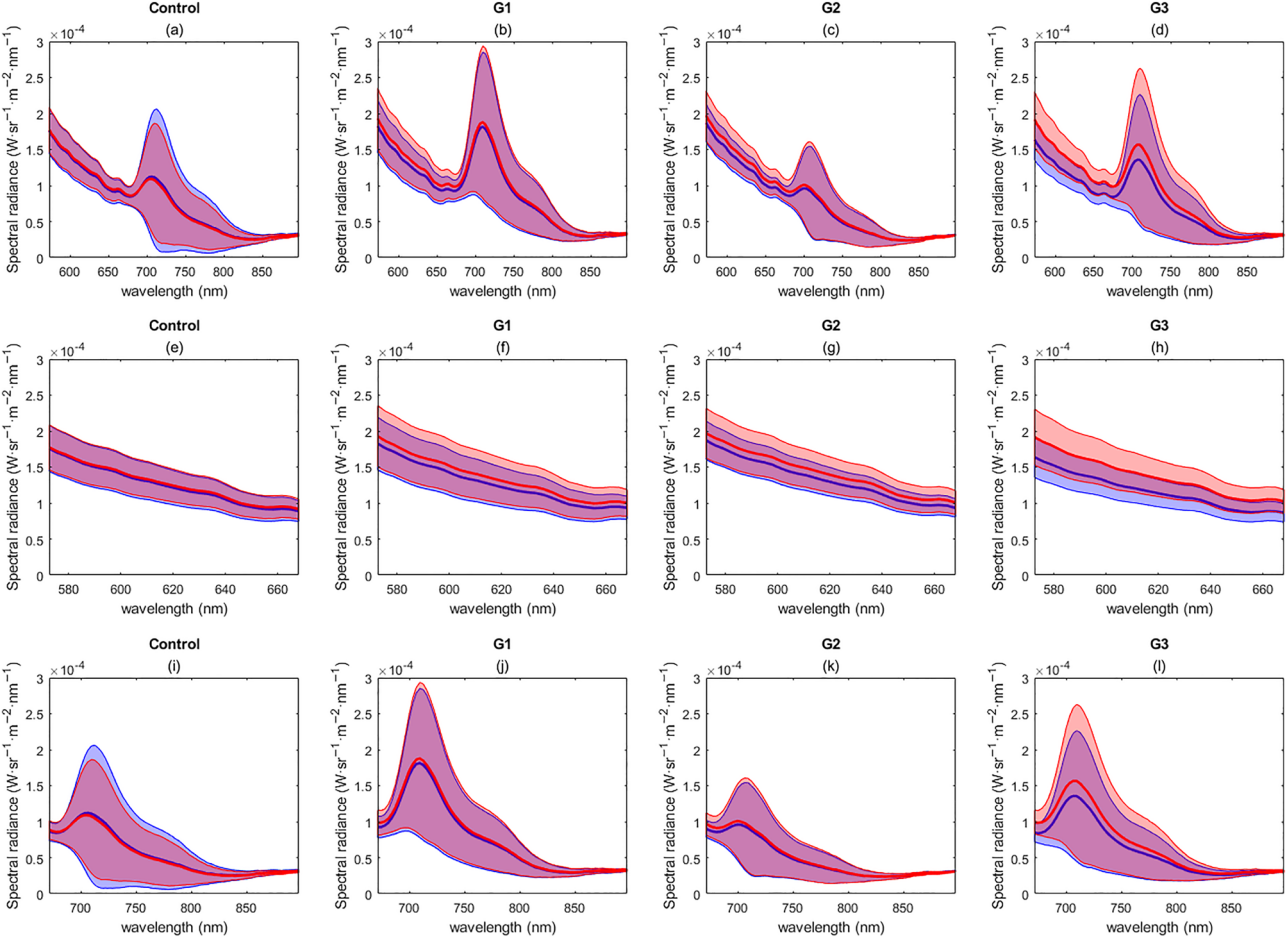
Average fluorescence emission spectrum of lumpfish per experimental group: (**a**–**d**) full spectral range, (**e**–**h**) green spectral range, (**i**–**l**) red spectral range. The experimental groups are the control group and the replicates (G1, G2, and G3). The figure represents the mean spectral signature (solid line) and the standard deviation (shade). The different colors represent the fluorescence spectra before the treatment (blue) and after the treatment (red).

Lumpfish fluorescence before and after the application of the therapeutic stressor was compared. The experimental groups consisted of three replicate groups receiving treatment (G1, G2, and G3), and a control group. The mean and the standard deviation (SD) of the fluorescence spectra (570–800 nm) for the experimental groups were classed as a combined single average of the fluorescence spectra (Fig. 3a–d). Substantial differences both between and within groups were found, suggesting the presence of individual-level variability in the biofluorescence patterns in lumpfish. To mitigate potential biases arising from differences between the groups, we analyzed the data separately for each group (composed of the same individuals) and compared the changes in biofluorescence patterns before and after treatment. The mean emission spectra differed between groups, particularly the red spectra between 690 and 750 nm (Fig. 3e–h). Additionally, there is a high overlap between the SD of the fluorescence spectra before and after the treatment for all the experimental groups (Fig. 3).

When examining the differences in the emission fluorescence spectra for each group, we observed a general trend of a similar mean and a highly overlapped SD before and after treatment. However, we did notice some differences in the replicates (G1, G2, and G3) after therapeutic treatment in the green spectral range, where there was a subtle increase in the mean and a lower overlap in the SD (Fig. 3f–h). This same trend was observed for G3 in the entire spectral range (Fig. 3d,h,l). Despite these observations, the information derived from the mean and SD emission fluorescence spectra (Fig. 3) is insufficient to provide evidence of relevant differences before and after treatment within each group.

**Figure 4:**
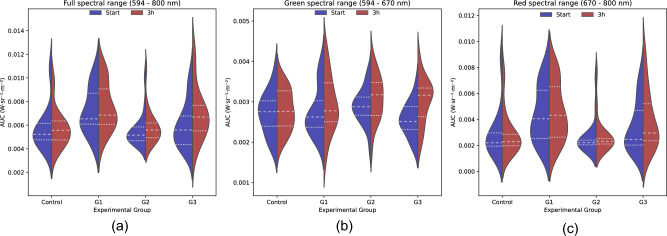
Violin plot showing the area under the curve (AUC) of the fluorescence spectra for the different experimental groups before (blue) and after (red) treatment. The experimental groups are the control group and the replicates (G1, G2, and G3). (**a**) Full spectral range, (**b**) Green spectral range, (**c**) Red spectral range.

The area under the curve (AUC), which is the radiance over the spectral range, of the fluorescence spectra, was calculated for an alternative quantification of fluorescence emissions. Violin plots were used to represent their distribution before and after treatment (Fig. 4). Considering the whole spectral range (594–800 nm), different distributions of the AUC between replicates (n = 3) were observed (Fig. 4a). Additionally, the distribution of the data corresponding to the control group, G2, and G3 presented a main peak but a long tail for the AUC high values. The AUC distribution changed in the green spectral range (594–670 nm) after treatment for all groups (Fig. 4b). Additionally, a bimodal distribution can be observed for G1. The violin plots in the red spectra (670–800 nm) did not reveal any noticeable differences in the AUC before and after treatment (Fig. 4c).

### Florescence type analysis

**Figure 5:**
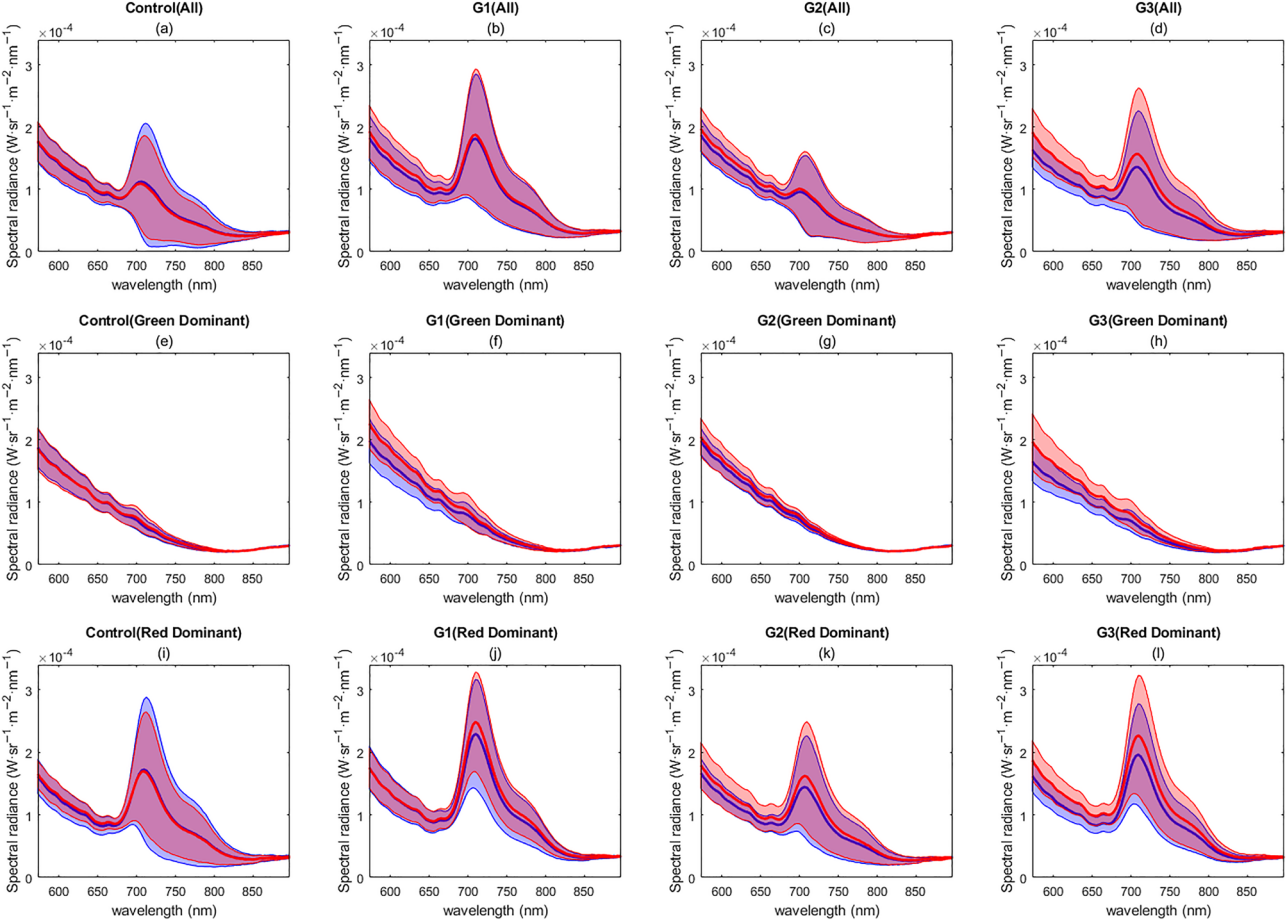
Average fluorescence emission spectrum of lumpfish per experimental group: (**a**–**d**) all lumpfish cohort together, (**e**–**h**) green dominant lumpfish, (**i**–**l**) red dominant lumpfish. The experimental groups are the control group and the replicates (G1, G2, and G3). The figure represents the mean spectral signature (solid line) and the standard deviation (shade). The different colors represent the fluorescence spectra before the treatment (blue) and after the treatment (red).

The analysis of the fluorescence signals before and after treatment can be influenced by the presence of different fluorescence emissions in the population. These distinct fluorescence emissions (green dominant and red dominant) showed significant variations in their signals. Therefore, to minimize the impact of individual-level variability in biofluorescence patterns, we conducted independent analyses for red dominant and green dominant lumpfish. This was done to prevent any potential biases that may arise from the mixing of individuals exhibiting different fluorescence signals. The mean and the SD of the spectra for the entire cohort (Fig. 5a), green dominant subgroup (Fig. 5b), and red dominant subgroup (Fig. 5c) are shown in Fig. 5. The SD of the fluorescence spectra for the two fluorescence types was found to be broadly overlapped before and after treatment. This overlap can be attributed to the analysis of samples at a group level and variability on the individual level in the biofluorescence patterns in lumpfish, as the lack of identification of specific individuals in the trial did not allow for individual-level analysis. As a result, the quantification of the differences in mean fluorescence spectra should be considered descriptive, without statistical relevance.

For green dominant samples, a difference in the mean fluorescence spectra can be observed, with fluorescence showing an increase in the mean spectral radiance after treatment of 11%, 4% and 16% for groups G1, G2, and G3, respectively (Fig. 5b) while the variations in the control group remained overlapped (1% increase). A similar trend was observed for red dominant samples, with an increase of 5%, 10%, and 15% in the measured mean spectral radiance for groups G1, G2, and G3, and a 1% increase for the control group. The mean fluorescence spectra were higher after treatment for all groups in the spectral range from 690 to 750 nm (Fig. 5c). Groups G1 and G3 also showed differences in the mean fluorescence spectra between 570 and 690 nm. There were no visible differences between the fluorescence after treatment in the control group.

#### Green dominant fluorescence

**Figure 6:**
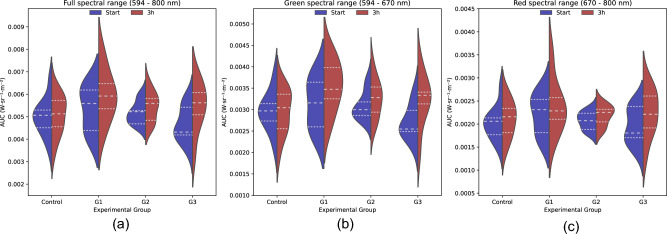
Violin plot showing the area under the curve (AUC) of the fluorescence spectra for the green dominant lumpfish per experimental groups before (blue) and after (red) treatment. The experimental groups are the control group and the replicates (G1, G2, and G3). (**a**) Full spectral range, (**b**) Green spectral range, (**c**) Red spectral range.

The violin plots for the green dominant samples showed a change of distribution and an increase in the fluorescence AUC for the groups after the treatment when the entire spectral range was considered (Fig. 6a). The change in the AUC distribution after treatment was more distinctive in the green spectra (594–670 nm) (Fig. 6b). These differences were more pronounced for G3, which exhibited higher mean and SD values after treatment. In the red spectra (from 670 to 800 nm), the range of the AUC values was similar to those within the green spectra (Fig. 6c). An increasing trend in the fluorescence AUC was observed for the groups after treatment, which was higher in groups G2 and G3. In summary, individuals with a dominant green fluorescence exhibited an increase in emission signal following treatment. These differences were most prominent in the green region of the spectra for the groups under the therapeutic stressor. Moreover, it was possible to observe bimodal distributions for the green and the red spectral ranges, which suggests that there are also subgroups within the green dominant lumpfish.

#### Red dominant fluorescence

**Figure 7:**
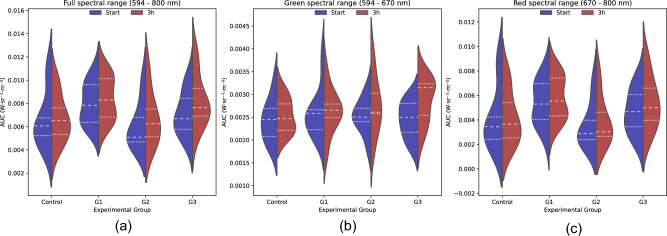
Violin plot showing the area under the curve (AUC) of the fluorescence spectra for the red dominant lumpfish per experimental groups before (blue) and after (red) treatment. The experimental groups are the control group and the replicates (G1, G2, and G3). (**a**) Full spectral range, (**b**) Green spectral range, (**c**) Red spectral range.

The violin plots of the red dominant subgroup for the entire spectral range showed a subtle increase in the AUC (Fig. 7a). The AUC distributions were calculated separately for the different spectral ranges (Fig. 7b,c), with higher contributions to the total AUC originated from the red spectra. However, significant changes after treatment were shown in the green spectra, where an increase in the mean fluorescence was observed for groups G1 and G3. The AUC distribution for the green spectra changed after the treatment period of 3 h for all replicates. Additionally, after treatment the AUC distributions for G2 and G3 shifted to a more asymmetric shape towards higher values. The difference in the red spectra AUC after the treatment of 3 h is not pronounced, which suggests that the main changes in fluorescence spectra after treatment for the red dominant subgroup were in the green spectra. In line with the findings for the green dominant subgroup, bimodal AUC distributions were observed for both green (control group) and red (G2) spectra, which also suggested the existence of subgroups within the red dominant lumpfish.

## Discussion

High differences in the average fluorescence spectra were observed between all experimental groups. We documented that the number of lumpfish per group showing variations in fluorescence (green dominant or red dominant) was different, with higher differences observed in the green spectra (594–670 nm). This variation in fluorescence may relate to the sex, dominance, or sexual maturity of the lumpfish within each experimental group. However, the lumpfish were randomly selected by an independent facility technician to prevent sex-based bias in the study. Hence, these different fluorescence types may influence the mean fluorescence spectra of the group under analysis. For this reason, we performed the same analysis independently for the different fluorescence signal types. Large individual variations of fluorescence signals were observed between the fish, and we found it reasonable to assume that the fluorescence in lumpfish depends on more than their reaction to the treatment. Documenting this variation produced by lumpfish has provided a contribution towards understanding the dynamics of fluorescence within this species. In retrospect, it would have been advantageous to track the individuals during the experiment and document other relevant information that could have contributed to explaining the behavior of the data. However, the welfare-based time constraint set for handling fish out of water was a limiting factor in our experimental design. The group method for assessing fluorescence was chosen as tracking individual fluorescence in an aquaculture setting with thousands of fish is not feasible with the technology available. As it stands, a group average seems inadequate for making a sound statistical inference on a treatment effect. This may be further influenced by the physiological tolerance of lumpfish to freshwater therapeutic baths received by the species in aquaculture. Finding a more precise method for tracking changes in fluorescence that matches the documented variation within lumpfish biology should be prioritized. The challenges related to the group average may be leveraged when using hundreds of individuals per trial, where the unexplained variation might be lower.

Nevertheless, this pilot study shows that lumpfish biofluorescence responds to a therapeutic stressor within a controlled experiment. Lumpfish are known to physiologically tolerate freshwater therapeutic baths well. As their tolerance to this treatment is typically higher than other fish species, the fact that a change in their baseline fluorescence levels can be observed from such a short-term impact means that their fluorescence responds quickly to their external stressors. This response was supported by the difference between the replicates and the control group. The mean fluorescence of the red spectra varies between these replicates, with lumpfish exhibiting varying intraspecific fluorescence. Such changes may be caused by individual male lumpfish exhibiting different levels of red fluorescence. However, the random selection of lumpfish did not involve sexing to simulate aquaculture conditions. Only G3 shows a higher red fluorescence emission after treatment than before. This may mean that the fluorophore emitting red fluorescence is manipulated by male lumpfish as seen in the analysis of lumpfish serum by Mudge and Davenport, 1986. Lumpfish serum sampled from both sexes contained a chromoprotein with a biliverdin prosthetic group; however, the male serum contained a red pigment such as phycoerythrin at a much higher rate than in females^7^. What is remains to be determined is whether fluorescence emissions produced in male lumpfish are systemic in nature or are concentrated and controlled within the skin chromatophores as found in the motile fluorescent organelles of the pygmy coral reef goby *Eviota pellucida*^23^ or the chromatophores of the Scorpaeniformes *Scorpaena maderensis* and *S. porcus*^24^.

Like in these Scorpaeniformes, it is documented that juvenile lumpfish regulate body pigmentation through chromatophores within minutes when background colours switch from dark to light^25,26^. However, our experiment showed that fluorescence is not controlled solely by chromatophores in lumpfish as the emission signals should be consistent between the replicates as well as the control. Additionally, the fluorescence emissions remained much more constant in the control group which underwent the same procedures as the experimental groups but in sea water. This supports our hypothesis that freshwater treatment causes observable changes in the fluorescence emissions spectra within the replicates. Observable color changes in teleost fish in response to stress have been noted in aquaculture conditions such as mackerel in sea cages, where digitally photographed bluer fish were associated with higher levels of plasma lactate^27^. However, clinical signs of stress such color changes already have biological costs at this point, such as reallocation of energy from growth to gluconeogenesis by the stress hormone cortisol and increased disease vulnerability through immune system suppression^28^. The increases in fluorescence emissions within the replicates in response to the therapeutic stressor were measured by hyperspectral imaging even though no observable external color changes were observed. Measuring these subclinical levels of stress may help the industry to systematically define culture conditions that the lumpfish would find least stressful.

The therapeutic freshwater bath given to the lumpfish should be considered stressful. For example, stress response in the channel catfish *Ictalurus punctatus* have been linked to the heme oxygenase (HO) breaking down hemoglobin causing increasing antioxidant biliverdin content when the animal is exposed to polycyclic aromatic hydrocarbons^29^. Biliverdin has been linked to the blue-green color in lumpfish^7,26,30^. Although fluorophores have not yet been isolated from lumpfish, bilirubin inducible fluorescent proteins responsible for biofluorescence in marine eels have been isolated from *Kaupichthys* eels (*Kaupichthys hyoproroides* and *Kaupichthys* n. sp.), *Anguilla japonica* and *Gymnothorax zonipectis*^31–33^. As biliverdin is transported within the serum, it circulates broadly within fish. The unique physiology of lumpfish may allow biliverdin levels and its associated fluorescence levels to change quickly. The bodies of gravid females are similar in density to seawater due to high water content within an extensive subcutaneous jelly and a loose-fibered dorsal musculature; mature males are similar but contain a higher lipid content^34,35^. This loosely structured, aqueous physiology may allow fluorophores to be transported quickly within the body.

In conclusion, several potential advantages of incorporating fluorescence level monitoring within lumpfish operations exist. This automated approach for monitoring lumpfish welfare includes increased sensitivity to subclinical changes within lumpfish, assessing stress levels on a large scale within production cohorts, and producing quantitative data that can help producers make informed husbandry decisions. Validation through further testing is needed to determine the feasibility of integrating these results into the existing welfare auditing programs for aquaculture operations raising lumpfish. The next step(s) for further developing a non-invasive parameter is to repeat the experiment with individually tagged fish to monitor variation in fluorescence through the therapeutic stressor. As the original hyperspectral imaging was designed to imitate the freshwater bath processing in an aquaculture setting where individual tagging is currently unfeasible, the pairwise design would be helpful to study how individuals respond to the treatment, both in terms of changes in fluorescence emission and its spatial distribution with the fish. The importance of individually analyzing lumpfish biofluorescence is also supported by our findings of bimodal distributions present within the AUC violin plots. This suggests the presence of subpopulations within the fluorescence subgroups (green dominant and red dominant). Furthermore, serum taken from lumpfish should be paired with hyperspectral imaging to measure biliverdin levels post treatment in both experimental and control groups. Proper identification of the fluorophore within lumpfish and finding its optimal excitation wavelength should be conducted during this period.

In terms of hyperspectral imaging, the development of algorithms should focus on areas that fluoresce the brightest, such as the tubercules on the dorsolateral lines. Once the dynamics of fluorescence emissions are linked to stress and can exclude chromatophore influenced changes as described in other biofluorescent scorpionfish, further tests would entail monitoring lumpfish responses to other stressors such as diet, density, or a pathogen. Underwater camera technology, imaging analysis programs, and artificial intelligence are rapidly making automated fish OWI monitoring a feasible alternative in aquaculture operations^36^. Biofluorescence monitoring in combination with other OWI monitoring standards can provide comprehensive, non-invasive monitoring within production units on a scale that has not been attempted to date. To the best of our knowledge, this is the first example of using hyperspectral imaging for monitoring biofluorescence as a potential welfare standard in aquaculture. Understanding stress in lumpfish prior to the manifestation of clinical signs has the potential to improve their welfare prior to the onset of either morbidity or mortality.

## Methods

### Experimental outline

A total of 3 replicates consisting of 20 lumpfish each was placed in a flow through 1000-L freshwater bath for 3 h on the same day, plus a control group (n = 20) who remained in saltwater for the same time period. The freshwater bath was chlorine free, oxygenated with an aeration system, and matched in temperature to the seawater containing the lumpfish (~ 6 °C). The control group received the same handling procedures as the experimental groups but were placed in salt water for the 3-h period. The lumpfish were imaged before and after the freshwater bath treatment with a hyperspectral camera as defined in Instrumentation.

### Lumpfish

The one cohort (same age and size class) of 80 lumpfish (931 ± 50 g, 27 ± 0.5 cm) utilized for this experiment were maintained at the Aquaculture Research Station located in Kårvik, Norway. The experiment was conducted on January 31st, 2023. Three groups of 30 fish were acclimated for 1 month in 3,500-L flow through tanks of the same light blue color supplied with filtered sea water. Tanks were maintained at ambient seawater temperatures (~ 6 °C) and salinity (36 psu) with 24-h lighting. Fish were starved for 24 h prior to utilization in the stress test. The experimental cohorts of lumpfish were humanely euthanized per regulatory requirements after the experiment using an overdose of the anesthetic benzocaine (Benzoak vet., ACD Pharmaceuticals AS, Oslo, Norway). The use of lumpfish for this experiment was authorized by the Norwegian Food Safety Authority (FOTS) ID 29929.

### Instrumentation

The hyperspectral imaging setup was a custom imaging platform consisting of a VNIR-1800 hyperspectral camera (Hyspex, Oslo, Norway) mounted above a conveyor belt with a custom illumination setup. The VNIR-1800 is a push-broom hyperspectral camera, which means that each frame consists of the spectral information of a thin spatial line across the field of view (x-axis) captured by this camera. The second spatial dimension (y-axis) was collected by spatially scanning the samples. The VNIR-1800 camera works in the Visible and Near Infrared (VNIR) spectral range (from 400 to 1000 nm), with a spectral resolution of 5.5 nm, and 1800 spatial pixels. The working distance between the camera and the samples was 100 cm, resulting in a field of view of 30.6 cm across the conveyor belt (x-axis), with a spatial resolution of 0.17 mm. The light source used in this experiment was LED lighting (G5 XR30 Pro Radion, Ecotech, Bethlehem, PA, USA) and the excitation wavelength selected was royal blue(wavelength peak at ~ 445 nm)^36^. A high exposure time (7.52 ms) was configured for the hyperspectral measurements to increase the sensitivity, since as the fluorescence signal was expected to be weak, and not detectable at a lower exposure time. The spatial resolution in the y-axis on push-broom cameras depends on the exposure time and the speed of the conveyor belt, which was configured to 6 cm/s resulting in a spatial resolution of 0.45 mm. The lumpfish were placed in pairs on a white plastic tray partially filled with the current tank water on a conveyor belt for spatial scanning.

A digital single lens reflex (DSLR) camera (D5100 /Nikon AF-S 60 mm f/2.8G IF-ED Micro lens, Nikon, Minato City, Tokyo, Japan) was used for photographing lumpfish presented in Fig. 2. A seawater filled aquarium was illuminated under royal blue illumination (~ 445 nm) to document biofluorescence while the ambient light photographs were taken with cool white light. A yellow barrier filter (Tiffen 62DY15 62 mm Deep Yellow 12 Filter) was utilized when capturing RGB to mitigate reflected blue light.

### Hyperspectral analysis

A common practice for conventional hyperspectral image applications that use broadband light is conducting a flat field calibration. This involves using a material with high and known reflectance in the spectral range of interest to convert the input radiance into reflectance, while also correcting for the spectral response of the instrumentation^37,38^. A radiometric calibration was performed to produce spectral radiance values in units of W m^−2^ sr^−1^ nm^−1^, as there is no agreement on a standard procedure for calibrating fluorescence spectral data. The radiometric calibration was performed using the HySpex Rad v2.5 software (Norsk Elektro Optikk, AS, Oslo, Norway).

The calibrated hyperspectral data was loaded into the Breeze analytical software (Prediktera, Umeå, Sweden) to segment the lumpfish. The segmentation of the fish consisted of selecting a spectral channel (483 nm) near the excitation wavelength showing a high contrast between the background and the fish, followed by the manual identification of the contour of the fish. The mean spectra from 594 to 800 nm were extracted for each sample and processed using MATLAB 2022b and the Seaborn Python package^39^.

### Statistical analysis

The sample size was decided based on a power analysis for an ANOVA test assuming a small to medium Cohen’s d^40^. However, the distribution of the data made an ANOVA test unsuitable, so the decision was made to keep the statistics on a descriptive level.

## Data Availability

The data that support the findings of this study are available on request from the corresponding author.
